# Ecophysiological Trade-Off Strategies of Three Gramineous Crops in Response to Root Extracts of *Phytolacca americana*

**DOI:** 10.3390/plants13213026

**Published:** 2024-10-29

**Authors:** Xinyu Wang, Yuting Cao, Yefei Jin, Lifu Sun, Fangping Tang, Lijia Dong

**Affiliations:** School of Life and Environmental Sciences, Shaoxing University, Huancheng West Road 508, Shaoxing 312000, China; wxinyu090@163.com (X.W.); cyt18668836532@163.com (Y.C.); botany@usx.edu.cn (Y.J.); sunlifu@163.com (L.S.); tangfp1972@163.com (F.T.)

**Keywords:** allelopathy, invasive plant, Gramineae, growth-defense strategy, antioxidant enzymes

## Abstract

The invasive *Phytolacca americana* L. poses a significant threat to local agroforestry ecosystems due to its allelopathic toxicity. However, the ecophysiological response mechanisms of crops to allelochemicals remain unclear. This study investigated the seedling growth, physiological, and biochemical responses of three gramineous crops to the root extracts of *P. americana* and identified potential allelochemicals of the invader. The germination and seedling growth of three crops were inhibited by extracts differently, with high-concentration extracts causing more severe inhibition on seedling roots in hydroponic (>57%) than soil culture experiments (>18%). This inhibition may be related to representative secondary metabolites such as fatty acyls, alkaloids, and phenols. Despite the significant inhibition of high-concentration extracts on seedling growth, the levels of soluble sugar, soluble protein, and antioxidant enzymes increased synergistically. Under allelopathic stress, three species enhanced antioxidant enzyme activities and metabolite contents at the cost of reducing their shoot, root biomass, and root/shoot ratio. This may be an ecophysiological growth-defense strategy to bolster their resistance to allelopathy. Interestingly, transgenic rice exhibited greater sensitivity to allelochemicals than wild-type rice, resulting in more pronounced growth inhibition and increased levels of most metabolites and antioxidant enzymes. This study highlights the adaptive strategies of three gramineous crops to the allelopathy of invasive *P. americana*.

## 1. Introduction

In light of global change, plant invasion poses a serious threat to food security, ecological safety, and human health. The impacts of plant invasion on main crops have garnered increasing attention in recent years [1,2]. *Phytolacca americana* L. (Phytolaccaceae), originally native to North America, has become a widespread invader in China [3], seriously threatening native agricultural production and biodiversity [4,5]. Some researchers found that the invader exhibited significant negative allelopathy on plants in agroecosystems [6,7,8]. For instance, it inhibited the growth performance of corn by damaging their cells and genetic material [6]. However, the allelopathic effects and underlying mechanisms of *P. americana* on other main crops such as rice and wheat remain unclear.

Allelochemicals, derived from plant residue decomposition, root exudates, leachates, and volatiles, such as phenols, terpenes, and saponins, can directly or indirectly favor or inhibit neighboring plants [9,10]. In invasion ecology, allelopathic effects are a critical mechanism helping exotic plants to invade successfully. The novel weapons hypothesis suggests that some invasive plants can outcompete native species by releasing allelochemicals [11]. These “novel weapons” are often effective because native species have not evolved defenses against them [12]. Especially root exudates, residues, and tissue extracts derived from invasive plants may significantly affect the germination and seedling growth of local plants, which finally determines the renewal, development, and survival of plant populations [13]. Noticeably, the ecological and physiological significance of weed–crop/crop–weed interferences is increasingly paid attention due to the needs of agronomic management practices [14]. On the one hand, the inhibitory effects of weeds on crops generally dominate over stimulative ones [15]. On the other hand, as the risk of invasion increases, understanding the magnitude and direction of allelopathic effects on crops becomes crucial for managing invasive plants in an agricultural ecosystem [11,16,17,18,19]. Currently, investigating allelopathic effects using plant extracts is still one of the primary strategies for exploring the allelopathic potential of invasive plants [1,20,21,22,23]. However, the allelopathic effects of invasive plants on crops mainly involve growth inhibition. For example, the aqueous methanol extracts of invasive *Ludwigia decurrens* inhibited the growth of *Oryza sativa* (rice) [24]. Similarly, methanolic extracts from 30 Malaysian invasive weed species showed obvious allelopathic activities, affecting the seed survival rate and seedling growth of weedy rice [25]. In contrast, the physiological and biochemical process, as well as tradeoff relationships (ecological response) between growth, physiological, and biochemical traits, which could reflect the fitness of crops, have been largely ignored. Thus, it is necessary to explore the impact of invasive plant extracts on the germination and seedling growth of crops. This will help comprehensively uncover the fitness of crops to allelochemicals by determining their growth, physiological, and ecological responses.

The successful invasion of *P. americana* is proved to be in correlation with its ecological adaptability, photosynthetic niches, resource utilization, and allelopathy [5,7]. The stem and leaf extracts of *P. americana* show a significant inhibitory allelopathic effect on *Digitaria sanguinalis* (L.) Scop. and *Cassia mimosoides* L. during seed germination and seedling growth [7,8], and they can also significantly alter the growth, development, and physiological metabolic processes of some crops, such as carrots, corn, cotton, and broomcorn [7,8,26]. However, the allelopathic effects of its root extracts on crop growth is unclear. In general, plants communicate through chemical signals, which convey information about environmental threats, or even trigger defense mechanisms, allowing plants to coordinate responses and optimize their survival strategies [27]. Except for aboveground interactions, belowground communication can also strongly mediate plant–plant interactions through secondary metabolites [28]. Therefore, it is very necessary to explore allelopathy driven by the roots of invasive plants on crops.

*P. americana* shows remarkable environmental adaptability and features a robust root system, which significantly changes the soil environment in invaded areas [3], exerting significant allelopathic effects on some local plants [6,7,8,26]. Therefore, the roots of *P. americana* are presumed to have a significant impact on the growth and development of rice and wheat. Furthermore, the life cycle of the invader spans from spring to autumn in China, significantly overlapping with the growth phases of summer crops such as wheat and rice. Here, we proposed three hypotheses: (1) the seed germination and seedling growth of crops can be inhibited by allelochemicals, but the intensity of inhibition may be species-specific; (2) seedling physiological characteristics can be obviously altered; and (3) the plants can adopt some strategies to cope with the allelopathic environment. To test these hypotheses, we determined the effects of *P. americana* root extracts on the germination and seedling growth of widely cultivated, economically significant crops (wheat and rice) in both hydroponic and soil culture experiments. Concurrently, we investigated the chemical composition using widely targeted metabolomic analysis to identify the potential allelochemicals. In addition, considering that genetically modified crops often exhibit stronger resistance to abiotic stress and are more frequently used in agricultural practices [29], we also selected a transgenic crop to deepen our understanding of the allelopathic potential of *P. americana* on crops.

## 2. Results

### 2.1. The Chemical Composition of Root Extracts of P. americana

**Figure 1:**
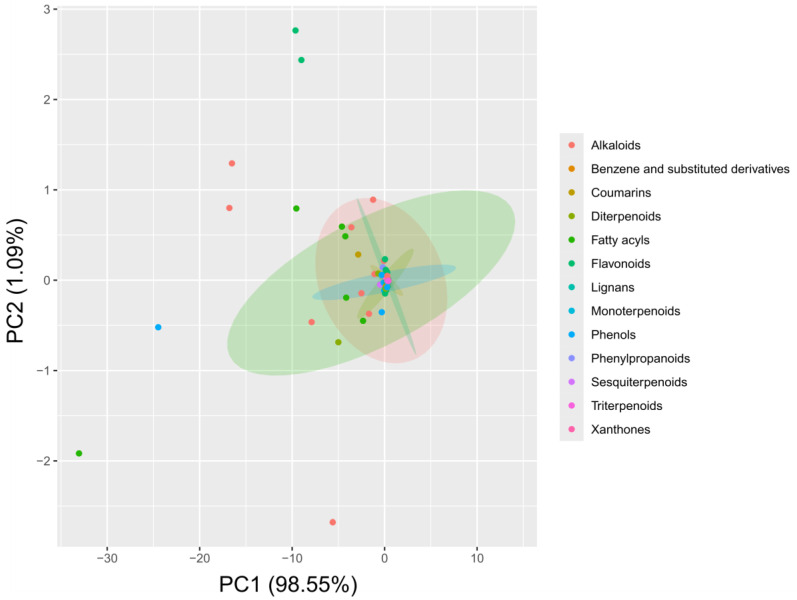
PCA analysis of major compounds from the root extracts of *Phytolacca americana*. The most representative compounds were fatty acyls, alkaloids, phenols, flavonoids, diterpenoids, and coumarins, respectively.

The allelochemicals generally originated from secondary metabolites [13], like alkaloids, fatty acyls, phenols, flavonoids, diterpenoids, and coumarins, so the non-secondary metabolites, including sugars, nucleotides, and nucleotide derivatives related to lipids and proteins were removed in PCA analysis (Figure 1 and Figure S1). The ion chromatogram of the quality control (QC) sample (Figure S2) exhibited symmetrical chromatographic peaks for all target compounds in the root extract of *P. americana*, which indicates an effective chromatographic separation of each target compound. The experimental and QC samples were densely clustered in the principal component analysis (Figure S3), suggesting that compound determination was robust and reliable.

**Figure 2:**
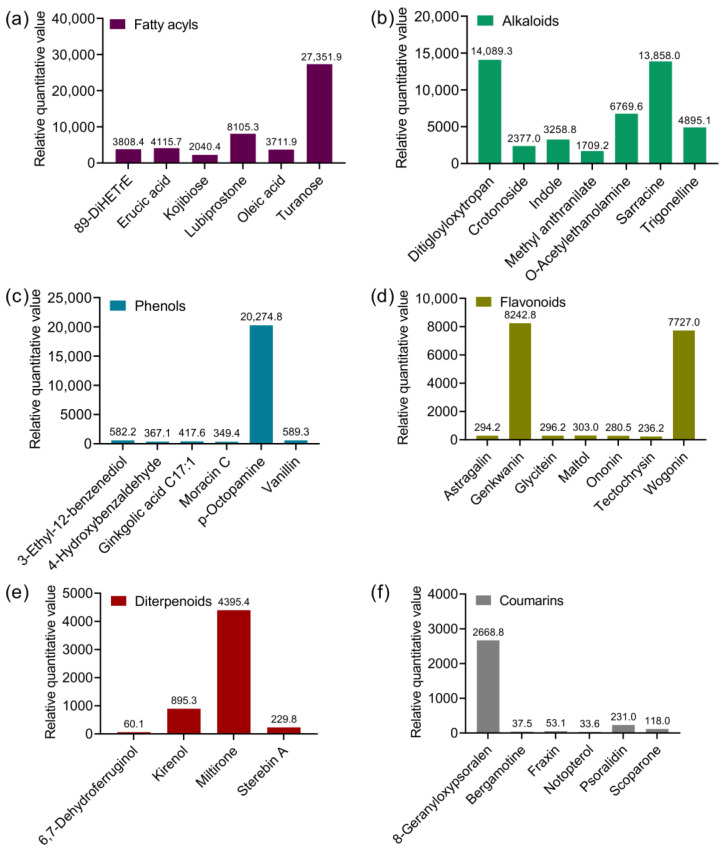
The relative quantitative value of representative compounds from root extracts of invasive *Phytolacca americana* (the proportion of each compound is greater than 1%). (**a**) Fatty acyls; (**b**) alkaloids; (**c**) phenols; (**d**) flavonoids; (**e**) diterpenoids; (**f**) coumarins. Ditigloyloxytropan indicates 3α,6β-Ditigloyloxytropan-7β-ol.

These compounds accounted for over 90% of the variation, indicating that the allelochemicals mainly originate from these substance categories (Figure 1). The fatty acyls, alkaloids, and phenols were the dominant categories (Figure 1). Herein, turanose, lubiprostone, 8,9-DiHETrE, erucic acid, oleic acid, and kojibiose were main compounds in fatty acyls; 3α, 6β-Ditigloyloxytropan-7β-ol, sarracine, O-Acetylethanolamine, and trigonelline were the main compounds in alkaloids; p-Octopamine was dominant in phenols; genkwanin and wogonin were dominant in flavonoids; and terpenoids and coumarins mainly contained Miltirone and 8-Geranyloxypsoralen, respectively (Figure 2a–f). Overall, the compounds with the highest relative quantitative values in the dominant category were turanose, p-Octopamine, 3α, 6β-Ditigloyloxytropan-7β-ol, and sarracine, respectively (Figure 2a–c).

### 2.2. The Allelopathic Responses of Three Crops in Hydroponic Experiment

**Figure 3:**
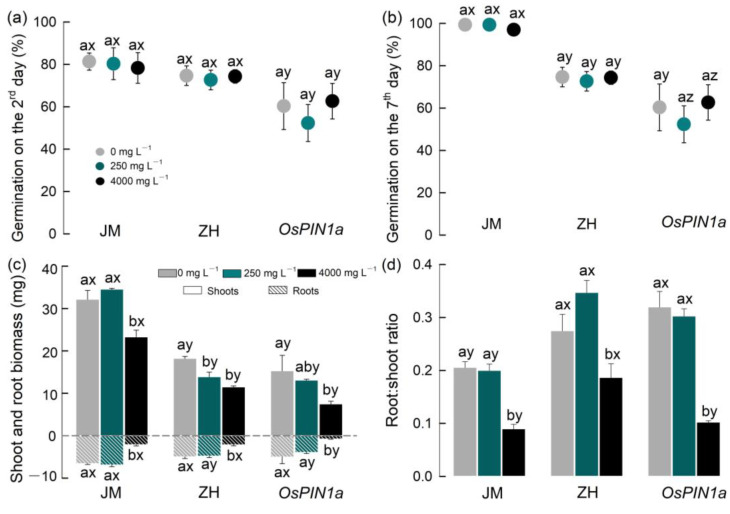
Effects of root extracts on the germination and growth of three gramineous plants in water culture experiment. (**a**) Germination on the second day; (**b**) germination on the seventh day; (**c**) shoot and root biomass; (**d**) root/shoot ratio. Lowercase letters (a, b) indicate the significant difference among the three concentrations of root extracts within each species, while the letters (x, y, z) indicate the significant difference among the three species (*p <* 0.05). All data are mean ± se, n = 3. JM: wheat; ZH: wild-type rice; *OsPIN1a*: *OsPIN1a* rice.

Both the interaction between species and extracts, and extracts exhibited no significant effect on the germination of the three crops by the 2nd and 7th days, with only interspecies differences proving significant (Table S2, Figure 3a,b). Specifically, the 7th day germination of wheat was significantly higher than that of rice (Figure 3b). The germination for the *OsPIN1a* transgenic rice at low and high extract concentrations (250 and 4000 mg·L^−1^) were 52.2% and 62.7%, respectively, obviously lower than that observed for wild-type rice at the same extract concentrations (72.7%, 74.6%, *p* < 0.001; Figure 3b).

From the 2nd to 7th day, the germination rate of wheat increased from approximately 80% to 100% across three extract concentrations. In contrast, the germination rate of both *OsPIN1a* transgenic and wild-type rice remained unchanged after the 2nd day (Figure 3a,b). Overall, although the germination speed of wheat was slower than that of rice, only the germination of *OsPIN1a* transgenic rice was significantly inhibited by extracts at concentrations of 250 and 4000 mg·L^−1^ (*p* < 0.05, Figure 3b) compared with the control.

In seedling growth, species and extracts significantly affected the aboveground and belowground biomass, as well as the root/shoot ratio, respectively (Table S3), while the interaction between species and extracts was significant only for the root/shoot ratio (Table S3). Specifically, compared with the control, the low-concentration extract only reduced the aboveground biomass of wild-type rice significantly (*p* < 0.05, −23.6%, Figure 3c), while the high-concentration extract significantly reduced the shoot and root biomass of the seedlings of all three crops (*p* < 0.01, Figure 3c). However, compared to the low-concentration extracts, high-concentration extracts significantly inhibited the shoot and root biomass of three seedlings, except for the shoot biomass of two rice species (Figure 3c). Among the three crops, the shoot and root of the transgenic rice were the most severely inhibited, with biomass reductions of 51.1% and 84.8%, respectively, which were greater declines than those observed in wheat (−27.6%, −68%) and wild-type rice (−36.8%, −57%) (Figure 3c).

Regarding the development of seedlings, roots displayed elevated sensitivity to allelochemicals compared to shoots. The root biomasses of wheat, wild-type rice, and *OsPIN1a* rice decreased by 68.0% (F_2_ = 69.271, *p* < 0.001), 57.0% (F_2_ = 19.500, *p* = 0.002), and 84.8% (F_2_ = 5.831, *p* = 0.039), respectively. These reductions were significantly larger than those observed in the corresponding shoot biomasses [−27.6% (F_2_ = 14.926, *p* = 0.005), −36.8% (F_2_ = 22.894, *p* = 0.002), and −51.1% (F_2_ = 3.474, *p* = 0.099)]. The root/shoot ratios of three seedlings followed a similar trend, with significant inhibition by the high-concentration extract (Table S3, Figure 3d).

**Figure 4:**
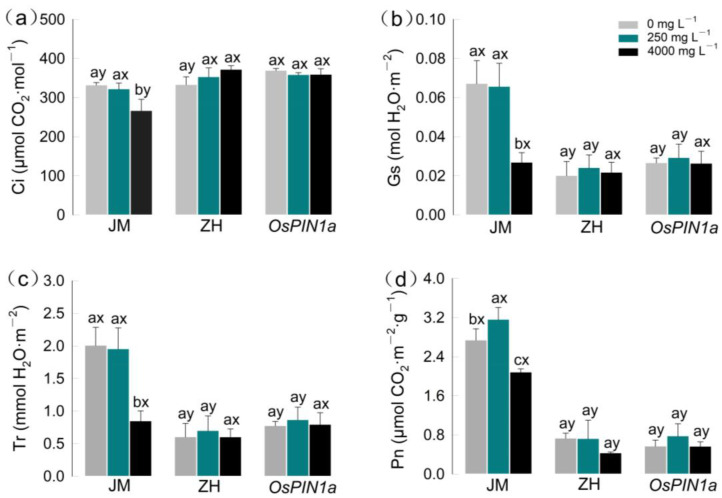
Effects of root extracts from *Phytolacca americana* on the photosynthetic indexes of three gramineous plants. (**a**) Gs; (**b**) Pn; (**c**) Ci; (**d**) Tr. Lowercase letters (a, b, c) indicate the significant difference among the three concentrations of root extracts within each species, while the letters (x, y) indicate the significant difference among the three species (*p <* 0.05). All data are mean ± se. JM: wheat; ZH: wild-type rice; *OsPIN1a*: *OsPIN1a* rice.

Species, extracts, and their interaction significantly affected the photosynthetic traits (Gs, Pn, Ci, Tr) of seedling leaves, with an exception for Ci not affected by extracts (Table S3). The photosynthetic traits in response to the extracts varied by species (Figure 4a–d): both wild-type and transgenic rice were unaffected by the concentration of the extract, with comparable photosynthetic indices (both *p* > 0.05); in contrast, wheat exhibited a significant response to the extract (*p* < 0.05, Table S3, Figure 4). The high-concentration extract reduced the Gs, Pn, Ci, and Tr of wheat leaves by 60%, 23.9%, 19.7%, and 57.9%, respectively, compared to those of the control (all *p <* 0.05), with reductions in Pn (F_2_ = 21.512, *p* = 0.002), Gs (F_2_ = 15.151, *p =* 0.005), and Tr (F_2_ = 18.307, *p* = 0.003) being particularly significant. The Gs, Ci, and Tr of wheat revealed no significant changes in the low-concentration extract (−2.2%, −3.0%, −2.7%), while Pn had a significant increase of 15.4% (*p* < 0.05). Furthermore, the Cs, Tr, and Pn of wheat under both control and low-concentration treatments were significantly higher than those of both rice species (Figure 4), aligning with the above-mentioned seedling biomass. Chlorophyll a and b content followed similar trends (Figure S4).

**Figure 5:**
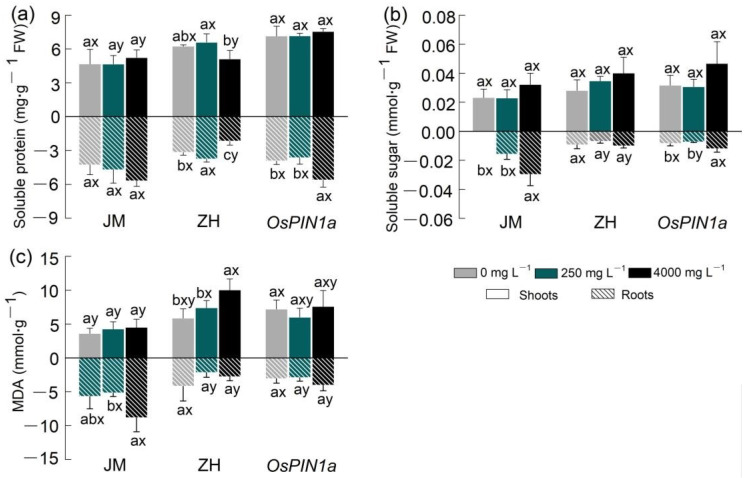
Effects of root extracts from *Phytolacca americana* on metabolites in the shoots and roots of gramineous plants. (**a**) Soluble protein content; (**b**) soluble sugar content; (**c**) MDA malondialdehyde. Lowercase letters (a, b) indicate the significant difference among the three concentrations of root extracts within each species, while the letters (x, y) indicate the significant difference among the three species (*p <* 0.05). All data are mean ± se. JM: wheat; ZH: wild-type rice; *OsPIN1a*: *OsPIN1a* rice.

The effects of extracts on the metabolites of the three species varied significantly except for soluble protein in shoots and roots (Table S2). However, there was significant interaction between extracts and species on the soluble protein in roots (Table S2, Figure 5). The soluble sugar in roots also showed a significant interaction between species and extracts (Table S2, Figure 5). Specifically, the low-concentration extract had a minimal impact; aside from a significant increase in soluble protein in the roots of wild-type rice (*p* < 0.05), the remaining plants showed no significant differences relative to the control (all *p >* 0.05, Table S2, Figure 5). The high-concentration extract affected three species to varying degrees (Figure 5): compared to the control, soluble sugar content in the roots of wheat increased significantly by 114.6% (*p* = 0.016), soluble protein content in the roots of wild-type rice decreased significantly by 31.4% (*p* = 0.005), MDA content in the shoot increased significantly by 46.8% (*p* = 0.010), and soluble protein and soluble sugar contents in the roots of transgenic rice increased by 43% (*p* = 0.007) and 42.1% (*p* = 0.035), respectively.

Regarding species-specific differences, soluble protein contents in the shoots and roots of transgenic rice seedlings treated with high-concentration extract were higher than those in wild-type rice seedlings (*p =* 0.001), while soluble sugar and MDA levels were comparable to those of wild-type rice seedlings (all *p >* 0.05). Soluble protein, soluble sugar, and MDA contents in the aboveground parts (shoots) of wheat were significantly lower than in those of rice (all *p <* 0.05); however, the contents of metabolites in the roots were higher than those in wild-type rice (all *p <* 0.05).

### 2.3. Changes in Antioxidant Enzyme Activities of Seedlings

**Figure 6:**
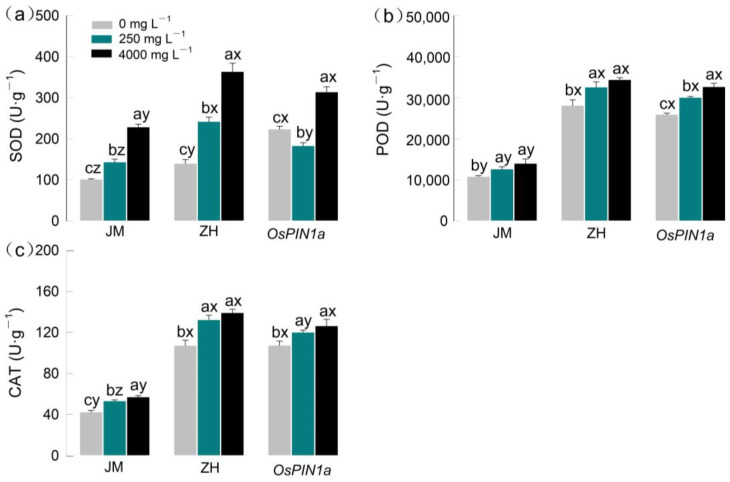
Effects of root extracts from *Phytolacca americana* on the leaf protective enzymes of three gramineous plants. (**a**) SOD; (**b**) POD; (**c**) CAT. Lowercase letters (a, b, c) indicate the significant difference among the three concentrations of root extracts within each species, while the letters (x, y, z) indicate the significant difference among the three species (*p <* 0.05). All data are mean ± se. JM: wheat; ZH: wild-type rice; *OsPIN1a*: *OsPIN1a* rice.

The antioxidant enzyme activities in the three species were significantly affected by allelopathic extracts, species, and their interactions (Table S3, Figure 6). Compared with the control, antioxidant enzymes (POD, CAT, SOD) increased significantly in all three plants under both low- and high-concentration extracts (all *p* < 0.05, Figure 6). However, the increase degree differed between low and high concentrations. Except for POD, CAT and SOD in wheat and wild-type rice exposed in low-concentration extract, which increased by 25.5%, 41.8% and 23.4%, 73.1%, respectively, all values were greater than those of *OsPIN1a*-type rice (11.9% and −18.2%, all *p* < 0.05, Figure 6). In high-concentration extract, POD, CAT, and SOD in wheat and wild-type rice seedlings increased by 29.8%, 34.9%, 126.1% and 22.3%, 29.9%, 160.3%, respectively, also being greater than in *OsPIN1a*-type rice seedlings (26.1%, 17.6%, and 40.4%; all *p* < 0.05; Figure 6). Especially for SOD, its increase was the most obvious: wheat (F_2_ = 340.012, *p* < 0.001), wild-type rice (F_2_ = 170.855, *p* < 0.001), and *OsPIN1a*-type rice (F_2_ = 136.728, *p* < 0.001).

### 2.4. The Pathway of Allelochemicals Affecting on Seedling Growth

**Figure 7:**
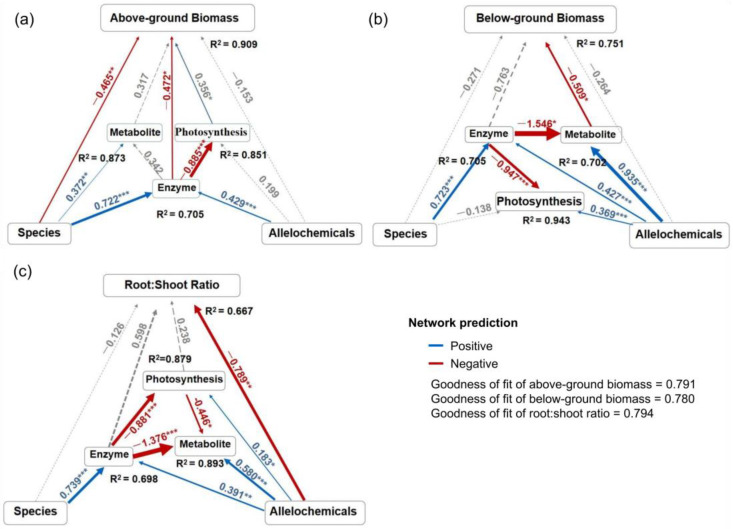
Path analysis model showing the effects of the allelochemicals (root extracts) from *Phytolacca americana* on the aboveground (**a**) and belowground biomass (**b**), and the root/shoot ratio (**c**) of gramineous plants. Red and blue arrows indicate significant negative and positive relationships, respectively. The solid and dash lines represent significant and non-significant relationships. The final models fit the data well, as assessed using goodness-of-fit and *R*^2^. * *p* < 0.05, ** *p* < 0.01, *** *p* < 0.001. The latent variables of enzyme activity contain the manifest variables: POD, CAT, SOD; photosynthesis: Ci, Gs, Tr, Pn; metabolite: soluble protein content, soluble sugar content, MDA malondialdehyde.

Although the growth of crops was significantly affected by the allelochemicals (root extracts) of *P. americana*, the aboveground and belowground parts of plants exhibited different responsive patterns, which also varied among species. The allelochemicals were expected to affect seedling growth directly or indirectly through physiological and biochemical processes, such as photosynthesis, enzyme activities, and metabolites. For shoots, species either directly or indirectly explained 41.46% of the variations in aboveground biomass (Table 1, Figure 7a). The allelochemicals in extracts inhibited the aboveground biomass primarily by indirectly increasing antioxidant enzymes (47.32% of variations) and reducing photosynthesis (34.47% of variations), but not the direct effect that accounts for only 5.89% of variations (−0.153, *p* > 0.05; Table 1, Figure 7a). In root growth, neither species nor extracts exhibited substantial direct effects, contributing to 13.28% and 25.77% of variations (−0.271, −0.246, both *p* > 0.05); oppositely, the influence path was indirect (Table 1, Figure 7b). In other words, the roots were indirectly inhibited due to changes in physiological and biochemical processes (−9.97% of variations for photosynthesis, 60.15% of variations for enzyme activities, and 10.64% of variations for metabolites). These changes were triggered by extracts (Table 1, Figure 7b). Notably, as extract concentration increased, both antioxidant enzyme activity and soluble metabolites in the seedlings first increased (0.427, 0.935, both *p* < 0.05). Then, the enzyme activity decreased the metabolites (−1.546, *p* < 0.05), which further inhibited the belowground biomass (−0.509, *p* < 0.05). Finally, the enzyme activity and metabolite indirectly inhibited root growth [(−0.509) × 0.935 + (−0.509) × (−1.546) × 0.427 = −0.140, Figure 7b]. Regarding the root/shoot ratio of seedlings, no association with the species was observed (Table 1, Figure 7c). Instead, the root/shoot ratio was directly determined by the allelopathic extracts, which accounted for 74.05% of the variation (Table 1, Figure 7c). The results indicate that as the concentration of allelochemicals increased, the allocation in roots significantly decreased.

### 2.5. The Allelopathic Responses of Three Crops in Pot Experiment

**Figure 8:**
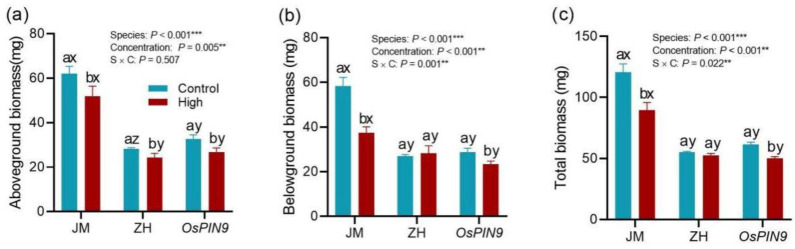
Effects of root extracts on the growth of gramineous plants in soil culture experiment; (**a**) aboveground biomass, (**b**) belowground, (**c**) total biomass. Lowercase letters (a, b) indicate the significant difference among the three concentrations of root extracts within each species, while the letters (x, y) indicate the significant difference among the three species (*p <* 0.05). All data are mean ± se (n = 5). JM: wheat; ZH: wild-type rice; *OsPIN1a*: *OsPIN1a* rice. ** *p* < 0.01, *** *p* < 0.001.

In the soil cultivation experiment, the aboveground, belowground, and total biomasses of the three seedling types were significantly affected by the high-concentration extract, species, and their interaction, except for the aboveground biomass (Figure 8). Overall, the extracts differently inhibited the growth of the three crop species (Figure 8). The inhibition effects for aboveground biomass were similar across the three species (F = 0.082, *p* > 0.05; Table S4, Figure 8a). However, the inhibition rates of belowground and total biomass for both *OsPIN1a*-type rice and wheat exceeded those of wild-type rice (F = 6.246, *p =* 0.014; F = 8.268, *p =* 0.005; Table S4, Figure 8b,c). These allelopathic effects of extracts were partially consistent with those observed in the hydroponic experiment: the inhibition effect on the roots was stronger in *OsPIN1a*-type rice compared to wild-type rice in both experiments (both *p* < 0.05; Figure 3c and Figure 8b).

## 3. Discussion

### 3.1. The Responses of Seed Germination and Seedling Growth to Allelopathic Extracts

Although the germination speed of wheat and rice differed, with rice germinating more quickly, only the germination of *OsPIN1a* transgenic rice was ultimately inhibited by extracts at two different concentrations. On the one hand, the embryo in rice seeds soaked for 48 h can develop rapidly when the seed absorbs sufficient water at an appropriate temperature. On the other hand, the allelochemicals may penetrate the seed coat differently, affecting the seed germination depending on the crop species [30]. Furthermore, in both hydroponic and soil cultivation experiments, the growth of all three seedlings was significantly inhibited by high-concentration extracts. This observation aligns with previous studies, which found *P. americana* exerted robust allelopathic inhibitory effects on the germination and seedling growth of common companion plants within its community, such as *C. mimosoides*, *Plantago virginica*, and *D. sanguinalis* [7,8]. Overall, the seedlings of three crops were more sensitive than seeds. Consistent with this, Zhang et al. [13] determined through meta-analysis that seedling growth is more sensitive to allelochemicals than seed germination. Hence, the seedling phase may be the most vulnerable and more susceptible to allelochemicals.

Three seedlings displayed significant variations in their allelopathic sensitivity. In the hydroponic experiment, the maximum inhibition rates for the shoot and root biomass of wheat, wild-type rice, and transgenic rice exposed to the high-concentration extract were 27.6% (68.0%), 36.8% (57.0%), and 51.1% (84.8%), respectively (Figure 3c,d). This variation could be attributed to differences in the tolerance of the three species to stress. Moreover, the increase in seedling photosynthesis, metabolism, and other physiological activities further confirmed this observed pattern. Intriguingly, the *OsPIN1a* transgenic rice seedlings exhibited more severe inhibition compared to the wild-type rice in both experiments. This suggests that while the transgenic rice showed robust growth and reproductive capabilities under standard soil conditions [31], it displayed a high level of sensitivity to allelopathic stress and is more prone to inhibitory effects. Importantly, under stress induced by allelochemicals, changes in root biomass among the three seedlings were significantly higher than those in shoot biomass in the hydroponic experiment, but this was not fully observed in the pot experiment. This implies that roots are more sensitive to allelochemicals than the shoot in hydroponic environments, but soil environments may alleviate the immediate damage on seedling roots to some degree [7,11,32,33]. However, the inhibition effects of high-concentration extracts on crop seedlings were obvious in either hydroponic or soil-cultured environments.

The inhibition effects on seedling growth and development were largely determined by allelochemicals. Kim et al. found that the concentrations of total phenolic compounds in the leaf extracts of invasive *P. americana* were 8–16 times higher than congeneric plants (exotic *P. esculenta* or native *P. insularis*), resulting in seed germination, and the seedling weight of these two congeneric plants were significantly inhibited [7]. Mikulic-Petkovsek et al. also found that flavonols, hydroxycinnamic acids, and stilbenes in *P. americana* were the major phenolic groups, which reduced 83% to 90% lower seed germination of perennial ryegrass [34]. However, the two studies just focused on the phenolic compounds but ignored other possible allelochemicals. Our metabolomic analyses revealed that these extracts primarily consisted of fatty acyls, alkaloids, phenols, flavonoids, diterpenes, and coumarins, which may be the potential categories of allelochemicals [35,36,37]. For example, the water and ethanol extracts of *Solidago canadensis* contain various allelochemicals such as terpenoids, fatty acids, flavonoids, and polyphenols [36,38]. Currently, related studies confirming all the allelochemicals of *P. americana* are extremely lacking. Here, although we did not completely confirm the specific chemicals functioning in allelopathy, the most abundant chemicals such as turanose, p-Octopamine, 3α, 6β-Ditigloyloxytropan-7β-ol, sarracine, genkwanin, and wogonin were presumed to play an important role in inhibiting the three crop seedlings, which will require more in-depth research in the future.

### 3.2. The Physiological and Biochemical Response of Seedlings to Allelopathic Extracts

Allelochemicals significantly altered the physiological metabolic activities of the three seedlings. Generally, allelochemicals affect photosynthesis by inhibiting/disrupting photosynthetic pigments or modifying the function of photosystem II (PSII) [30]. In this study, under the high-concentration extract treatment, the significant suppression of Gs, Pn, Ci, and Tr in wheat was observed, although the photosynthetic pigments remained largely unaffected. Therefore, we speculate that the function of PSII in wheat leaves was compromised [39]. This phenomenon has been observed in allelopathy studies. For example, the fatty acid allelochemical suppresses oxidation by PSII in leaves and thus inhibits the photosynthetic reaction [40,41]. In the root exudates of *P. americana*, the fatty acyls, as one of the dominant allelochemicals, may affect the photosynthetic reaction. However, responses vary among species: the photosynthesis of the two rice species showed no significant suppression of Gs, Pn, Ci, and Tr.

Regarding the metabolites in biochemical processes, the accumulation of osmoregulatory substances allows plant cells to regulate intracellular osmotic potential, maintain water equilibrium, and preserve the structure of normal cell membranes [30]. Although the roots of wheat and *OsPIN1a* rice were inhibited when they were being exposed to the extract, metabolites significantly increased in the roots. This attributes to a trade-off between cost and benefits. On the one hand, the production of secondary metabolites needs amounts of nutrients and energy; on the other hand, they can protect plants against the biotic and abiotic environment [37]. Similar patterns were also found: extracts derived from the shoot of chrysanthemum significantly inhibited its own root growth, but stimulated an increase in soluble sugar and protein levels in the roots [42]. Such ecophysiological responses may be an important strategy to mitigate the damage inflicted by allelochemicals.

As the allelochemical concentration increased, so did the antioxidant enzyme activity in the roots of the three species, which may be critical for seedlings to efficiently eliminate excessive ROS accumulated in the cells. Because, upon exposure to allelochemicals, plant cells rapidly generate reactive oxygen species (ROS), resulting in rapid changes in the activity of antioxidant enzymes such as POD and SOD to counteract oxidative stress [30,43]. Moreover, the antioxidant enzyme system is essential for maintaining reduced levels of free radicals in plant cells and protecting cell membrane lipids from ROS-induced damage. Hence, the synergistic action of POD, SOD, and CAT can effectively prevent ROS-induced structural and functional damage to biological membranes. Our results suggest that the three crop seedlings can modulate their antioxidant enzyme systems to varying extents under allelopathic stress, protecting the organism against damage. This may explain why seedling growth remained largely uninhibited under low-concentration extract exposure. When the concentration of extracts exceeds the defensive capacity of cellular antioxidant systems, growth inhibition occurs. Interestingly, the response of the antioxidant enzyme system varies among species: *OsPIN1a* transgenic rice exhibited the most significant increase in enzyme activities, followed by wild-type rice and wheat. This implies that *OsPIN1a* transgenic rice was more sensitive to allelopathic stress. This could be attributed to a higher expression of *OsPIN* genes and elevated negative phototropism in rice roots, which confers to the transgenic rice a stronger resistance to abiotic stress [44,45]. A similar phenomenon was also found in transgenic and wild-type Arabidopsis facing salt stress [46]. In total, three crop seedlings altered the physiological metabolic activities to cope with the stress of allelochemicals.

### 3.3. The Ecological Response of the Seedling to Allelopathic Extracts

In fact, the accumulation of plant biomass correlates strongly with the intrinsic metabolism, photosynthesis, and enzyme variations within a plant [2,30]. According to the path analysis, the factors affecting seedling growth included the antioxidant enzyme activities, plant species, and the extract concentration. The three factors changed in a synergistic fashion, resulting in the allelopathic response in the three crops’ seedlings. Aboveground biomass reduced when exposed to allelopathic stress, but its strength was strongly species-specific. Differences in belowground biomass were primarily caused by metabolic changes, which were mediated by both antioxidant enzymes and extract concentration. Noticeably, the root/shoot ratio of the three seedlings could be directly affected by allelochemicals, and the physiological changes including antioxidant enzyme activities and metabolites were indirect driving factors. These pathway analyses suggest that there were different allelopathic responses between aboveground and belowground parts for crop seedlings, which may be attributed to the divergent functions of the root and leaf that trigger different physiological responses to allelopathic stress [37]. When confronted with allelopathic stress, crop seedlings tended to adopt an ecophysiological trade-off strategy between growth and defense [2]. This strategy might represent the primary pathway underlying the allelopathic inhibitory effects of invasive plants.

## 4. Conclusions

Here, we examined the growth, physiological, and ecological responses of three gramineous crops to the root extracts of the invasive species *P. americana* through two experiments. We also conducted a preliminarily exploration of the allelochemicals involved. Our findings supported our three hypotheses. The allelochemicals from *P. americana* roots significantly inhibited the growth of the three seedlings, with the degree of allelopathy being species-specific. This variation in allelopathic strength may be attributed to certain allelochemicals, such as fatty acyls, alkaloids, phenols, flavonoids, diterpenes, and coumarins. Additionally, the physiological characteristics of the seedlings, including antioxidant enzyme activity and metabolite contents, were synergistically altered. Interestingly, the crop seedlings appeared to mediate their growth and physiological traits to counteract allelopathic stress.

## 5. Materials and Methods

### 5.1. Study Species and Extracts Preparation

Six sites invaded by *P. americana* were randomly selected near Changzhao Reservoir in Shaoxing City, Zhejiang Province (120°6′~120°26′ E, 29°26′~29°24′ N), and any two sites were at least 500 m apart. The average height and density of *P. americana* populations were 0.87 ± 0.10 m and 5.8 ± 0.6 m^−1^, and most individuals in each population were in the vegetative growth stage. The pH, organic carbon, total nitrogen, and available phosphorus of invaded soils were 6.87 ± 0.37, 34.97 ± 4.71 g·kg^−1^, 1.88 ± 0.20 g·kg^−1^, and 12.39 ± 1.69 mg·kg^−1^, respectively. The area was characterized by a subtropical monsoon climate, with an average annual precipitation of 1469.8 mm and an annual average temperature of 16.9 °C [National Meteorological Science Data Center, http://data.cma.cn (12 December 2021)].

Five *P. americana* individuals were then randomly selected and their roots were harvested and mixed as a sample. Thus, six root samples were acquired from six sites in total. To obtain the root extracts of *P. americana*, each root sample (50 g) was dried at 40 °C, crushed, and passed through a 50-mesh sieve. Then the sieved powder was soaked in 150 mL of 80% ethanol solution. After 24 h, the solution was filtrated, and the filtrate was concentrated in a rotary evaporator to obtain six pasty root extracts. All the experiments were conducted at Shaoxing University.

To further identify root metabolites in *P. americana*, a widely targeted metabolomic analysis based on UPLC-MS/MS was performed by Biotree Biotechnology Co., Ltd., (Shanghai, China). Specifically, the six freeze-dried roots were ground, respectively. Then, 50 mg sample powder from each root sample was extracted with 700 µL of methanol-water (75:25, *v*/*v*) containing external standard. After filtration, the filtrate was collected for further analysis. The UPLC separation was carried out on a Waters Acquity HSS T3 column (100 × 2.1 mm, 1.8 μm) using an EXIONLC System (Sciex, Framingham, MA, USA). The mobile phases consisted of 0.1% formic acid in water (A) and acetonitrile (B). The column temperature was set at 40 °C. The gradient elution program was set as follows: 0−0.5 min, 2% B; 0.5−10 min, 2−50% B; 10−11 min, 50−95% B; 11−13 min, 95% B; 13−13.1 min, 95−2% B; 13.1−15 min, 2% B. The flow rate was set at 0.40 mL/min. The auto-sampler temperature was set at 4 °C, and the injection volume was 2 μL. A Sciex QTrap 6500+ (Sciex Technologies, Framingham, MA, USA) was applied for assay development. Typical ion source parameters were as follows: ion spray voltage, +5500/−4500 V; curtain gas, 35 psi; temperature, 400 °C; ion source gas, 1:60 psi; ion source gas 2, 60 psi; DP: ±100 V. SCIEX Analyst Work Station Software (Version 1.6.3) was employed for MRM data acquisition and processing. In-house R program and database were applied to peak detection and annotation.

### 5.2. Hydroponic Experiment

To evaluate the allelopathic potential, three concentration gradients of 0 mg·L^−1^, 250 mg·L^−1^, and 4000 mg·L^−1^ of root extracts were set up, according to a previous study, which were identified as critical levels that impact native species and crop seedlings [8]. Three extracts were randomly selected from the above-mentioned six extracts as 3 replicates and thus it was a total of 9 combinations (3 extracts × 3 species) and 3 replicates. In the meta-analysis of Zhang et al. [13], plant leachate was usually between 20~10^6^ mg·L^−1^. Thus, the concentration used here fell into the lower chemosensitivity range. Furthermore, three common gramineous crops were selected to suggest their responses to the root extract, i.e., wheat (Lufeng Jimai 22), wild-type rice (Zhonghua 11), and transgenic rice (overexpression of growth hormone polar transport output vector *OsPIN1a*, abbreviated as *OsPIN1a*). Herein, Jimai 22 is highly yielded and the most popular in China’s Huanghuai and north eastern areas [47]. The Zhonghua 11 is a widely used wild-type rice in various agronomic studies due to its well-characterized genome and stable traits [48]. The *OsPIN1a* gene is involved in the morphogenesis of various organs in rice, and its expression is substantially higher in roots, leaves, and juvenile spikes than in wild-type rice [29].

All seeds were sterilized with 1% KMnO_4_, immediately rinsed, and soaked for 48 h with the equivalent concentrations of root extracts or water (control). Then, 50 uniformly sized seeds were selected and placed in each germination box (15 cm in diameter and 5 cm in height) with sterilized wet gauze for germination and seedling growth. The experiment was conducted in an artificial climate chamber with 12/12 h day/night and temperatures of 28/20 °C, from 15 March to 10 April 2022. To minimize positional effects, germination boxes were randomly positioned, and germination was tested on the 2nd and 7th day. After 7 days, the original culture solution was replaced with Hoagland nutritional solution containing the corresponding concentrations of allelopathy extracts, and seedling growth continued until the 25th day. During seedling growth, the solution was supplied in time and all other conditions were kept constant. Before the plants were harvested, five individuals in each box were randomly selected and the third leaf in each individual was selected to test photosynthetic indicators such as net photosynthetic rate (Pn), transpiration rate (Tr), intercellular CO_2_ concentration (Ci), and stomatal conductance (Gs) using a Li-6400 portable photosynthesizer (LI-COR, Lincoln, NE, USA). The average value of five individuals for each photosynthetic trait were obtained in each treatment. After all the seedlings were harvested, both the fresh root and shoot of each plant were weighted. Then they were divided into two sections: (1) the partial one (five individuals) was weighted again and then dried at 65 °C for 48 h to acquire shoot and root biomass, which could help us calculate the biomass of all the seedlings; (2) the other seedlings were flash-frozen in liquid nitrogen and stored at −80 °C for further biochemical (enzymes, metabolites) tests.

A total of 100 mg of fresh leaf in each treatment was extracted with aqueous 80% acetone and the suspension was filtered through a Whatman filter paper No. 1. Chlorophyll contents were determined spectrophotometrically using spectronic GENESYS 20 spectrophotometer (Thermo Electron Corporation, Waltham, MA, USA) at 2 wavelengths: 663 nm for chlorophyll a and 647 nm for chlorophyll b. The contents were calculated using Lichtenthaler’s equation and expressed as mg·g^−1^ dry weight [49].

Allelochemicals can often affect the balance of the antioxidant system and related enzymatic activity [42,49]. Thereby, we measured the peroxidase (POD), superoxide dismutase (SOD), and catalase (CAT) in leaves of seedlings using corresponding kits (Nanjing Jianjian Bioengineering Institute, serial numbers A084, A001, and A007, respectively; see http://www.njjcbio.com/). In addition, Malondialdehyde (MDA) and soluble sugar content in both shoots and leaves were determined by the thiobarbituric acid (2-thiobarbituric acid) method, and soluble protein concentration in both shoots and leaves was assessed by Caulmers Brilliant Blue G-250 colorimetric technique [42].

### 5.3. Soil Culture Experiment

To further evaluate the allelopathy potential of root extracts on seedling growth of the three crops in the soil environment, the root extracts with similar concentrations were added to 1 L pots containing 850 mL sterilized soil in a gradient of 0 mg·L^−1^, 4000 mg·L^−1^, and the three gramineous crop species were grown on 15 May 2022. Here, because of the more significant inhibition effects of extracts in the hydroponic experiment, we did not test the effects of low-concentration extracts. The soil properties such as pH, organic carbon, total nitrogen, and available phosphorus were 7.44, 25.65 g·kg^−1^, 1.23 g·kg^−1^, and 5.25 mg·kg^−1^, respectively. Each treatment was repeated five times for a total of thirty pots. Briefly, the seed soaking method was as above, and then the seeds were sown in the pots. After 60 days, the seedlings were collected, dried at 60 °C and weighed to obtain root and shoot biomass.

### 5.4. Data Analysis

In metabolome analysis, the mass spectrometry data and quantitative analysis of all molecules were performed using the SCIEX Analyst Work Station Software (Version 1.6.3). All the other germination and growth data were analyzed using R v4.4.1 (R Core Team., 2024) [50]. The main chemical species were screened by the principal component analysis (PCA) using “ggplot2” and “factoextra” packages. The plant growth and physiological traits in several treatments were investigated using two-factor ANOVA and LSD multiple comparisons (“emmeans” package). The inhibition effects of extracts among three species were analyzed through one-way ANOVA and LSD multiple comparisons. Partial least squares path modeling (PLS-PM) was performed to quantify the direct and indirect of root extracts on seedling growth through the physiological and biochemical traits using the “plspm” and “devtools” packages. Furthermore, we quantified the relative importance of each physiological index on plant growth as follows:

$$Importance=\frac{\sum_{j}\beta_{j}cor(y,xj)}{R^{2}}\times 100\%$$

*β* denotes the direct path coefficient. y and x denote the potential dependent and independent variables, respectively. *j* denotes the number of potential independent variables explaining the potential dependent variable. *R*^2^ denotes the total explanation of the potential dependent variable.

## Figures and Tables

**Table 1:** Contributions to *R*^2^ of crop species, allelochemicals (root extracts), and physiological traits to crop seedling growth by three path models.

	Latent Variables	Path Coefficients	Correlation	Contribution to *R*^2^ for Each Variable (%)
Aboveground biomass	Species	−0.465	−0.811	41.46
	Allelochemicals	−0.153	−0.350	5.89
	Photosynthesis	0.356	0.880	34.47
	Enzyme	−0.472	−0.912	47.32
	Metabolite	0.317	−0.835	−29.14
Belowground biomass	Species	−0.271	−0.368	13.28
	Allelochemicals	−0.264	−0.733	25.77
	Photosynthesis	−0.217	0.345	−9.97
	Enzyme	−0.763	−0.592	60.15
	Metabolite	−0.509	−0.157	10.64
Root/shoot ratio	Species	−0.126	0.341	−6.44
	Allelochemicals	−0.789	−0.626	74.05
	Photosynthesis	0.238	−0.301	−10.74
	Enzyme	0.598	0.209	18.74
	Metabolite	−0.283	−0.575	24.40

## Data Availability

The data presented in this study are available on request from the corresponding author. The data are not publicly available due to privacy.

## Supplementary Materials

The following supporting information can be downloaded at: https://www.mdpi.com/article/10.3390/plants13213026/s1. Figure S1: The ion flow in the extraction of quality samples. Figure S2: The score of PCA. Green dots represent quality control samples, and blue dots represent experimental samples. Figure S3: PCA analysis of total compounds from root extracts of invasive *Phytolacca americana*. Figure S4: The contents of chlorophyll a (A) and b (B) of three gramineous plants. Table S1: The mobile phase conditions of liquid chromatography. Table S2: Two-way ANOVA analysis for effects of *Phytolacca americana* extracts on the germination and biochemical characters of three gramineous crops in hydroponic experiment. Table S3: Two-way ANOVA analysis for effects of *Phytolacca americana* extracts on the growth and partial physiological characters of three gramineous crops in hydroponic experiment. Table S4: One-way ANOVA analysis for the inhibition percentage of high-concentration extracts on the growth of three gramineous crops in pot experiments.
