# Supplementary material for: Ecophysiological Trade-Off Strategies of Three Gramineous Crops in Response to Root Extracts of Phytolacca americana
Source: Plants (Basel). 2024 Oct 29;13(21):3026. doi: 10.3390/plants13213026 (PMC11548188; doi:10.3390/plants13213026)
Supplement: Supplementary file 1 [file plants-13-03026-s001.zip › plants-3199621-supplementary.pdf]

Ecophysiological trade-off strategies of three gramineous crops in response to root extracts of *Phytolacca americana*

Xinyu Wang, Yuting Cao, Yefei Jin, Lifu Sun, Fangping Tang, Lijia Dong*

School of Life and Environmental Sciences, Shaoxing University, Huancheng West Road 508, Shaoxing, 312000, China.

*Author for correspondence: School of Life and Environmental Sciences, Shaoxing University, Huancheng West Road 508, Shaoxing, 312000, China.

Email: Donglijia@126.com

Running title: An ecophysiological response of crops to invasive allelopathy

Table S1 The mobile phase conditions of liquid chromatography.

Time (min)	Velocity of flow (μL/min)	A% Water	B% Acetonitrile
0	400	98	2
0.5	400	98	2
10	400	50	50
11	400	5	95
13	400	5	95
13.1	400	98	2
15	400	98	2

Table S2 Two-way ANOVA analysis for effects of *Phytolacca americana* extracts on the germination and biochemical characters of three gramineous crops in hydroponic experiment. *$P < 0.05$, **$P < 0.01$, ***$P < 0.001$.

		species	extracts	species*extracts
The 2 rd germination	df	2	2	4
	F	22.365***	0.745	0.620
	P	< 0.001	0.489	0.654
The 7th germination	df	2	2	4
	F	101.183***	0.886	0.889
	P	< 0.001	0.430	0.490
Soluble protein in shoots (mg·g^{-1})	df	2	2	4
	F	26.094***	0.137	2.163
	P	< 0.001	0.873	0.115
Soluble sugar in shoots (mmol·g^{-1})	df	2	2	4
	F	3.873*	5.541*	0.408
	P	0.040	0013	0.800
MDA in shoots (mmol·g^{-1})	df	2	2	4
	F	16.413***	4.249*	1.888
	P	< 0.001	0.031	0.156
Soluble protein in roots (mg·g^{-1})	df	2	2	4
	F	22.536***	3.074	7.707**
	P	< 0.001	0.071	0.001

Soluble sugar in roots	df	2	2	4
(mmol·g^{-1})	*F*	25.617***	10.896**	3.873*
	P	< 0.001	0.001	0.020
MDA in roots (mmol·g^{-1})	df	2	2	4
	F	21.245***	4.498*	2.733
	P	< 0.001	0.026	0.061

Table S3 Two-way ANOVA analysis for effects of *Phytolacca americana* extracts on the growth and partial physiological characters of three gramineous crops in hydroponic experiment. *$P < 0.05$, **$P < 0.01$, ***$P < 0.001$.

		species	extracts	species*extracts
Above-ground	df	2	2	4
biomass (mg)	*F*	114.089***	20.698***	2.193
	P	< 0.001	< 0.001	0.111
Below-ground	df	2	2	4
biomass (mg)	*F*	7.977**	38.369***	1.172
	P	0.003	< 0.001	0.356
Root:shoot ratio	df	2	2	4
	F	21.737***	55.272***	3.733*
	P	< 0.001	< 0.001	0.022
POD ($U \cdot g^{-1}$)	df	2	2	4
	F	1329.929***	90.322***	4.126*
	P	< 0.001	< 0.001	0.015
CAT ($U \cdot g^{-1}$)	df	2	2	4
	F	997.003***	75.141***	4.652**
	P	< 0.001	< 0.001	0.009
SOD ($U \cdot g^{-1}$)	df	2	2	4
	F	190.486***	449.068***	44.053***
	P	< 0.001	< 0.001	< 0.001

Pn (μmol·m^{-2}·s^{-1})	df	2	2	4
	F	298.188***	15.280***	4.603*
	P	< 0.001	< 0.001	0.010
Gs (mol·m^{-2}·s^{-1})	df	2	2	4
	F	42.608***	9.878***	8.514***
	P	< 0.001	0.001	< 0.001
Ci (μmol·mol^{-1})	df	2	2	4
	F	28.265***	1.575	8.096***
	P	< 0.001	0.234	0.001
Tr (mmol ·m^{-2}·s^{-1})	df	2	2	4
	F	53.658***	11.016**	9.083***
	P	< 0.001	0.001	< 0.001

Gs, stomatal conductance; Pn, net photosynthetic rate; Ci, intercellular CO_2 concentration; Tr, transpiration rate; POD, peroxidase; CAT, catalase; SOD, superoxide dismutase. *P < 0.05, **P < 0.01, ***P < 0.001.

Table S4 One-way ANOVA analysis for the inhibition percentage of high-concentration extracts on the growth of three gramineous crops in pot experiments. All data are mean ± se. Lowercase letters (a, b, c) indicate the significant difference among three species.

	wheat	Wild-type rice	Transgenic rice (*OsPIN1a*)

Above-ground biomass (g)	-16.31 ± 7.19% a	-14.17 ± 7.16% a	-18.03 ± 5.81% a
Below-ground biomass (g)	-35.81 ± 4.48% a	4.9 ± 13.51% c	-18.92 ± 4.98% ab
Total biomass (g)	-25.76 ± 5.23% a	-4.84 ± 2.98% c	-18.44 ± 2.16% ab

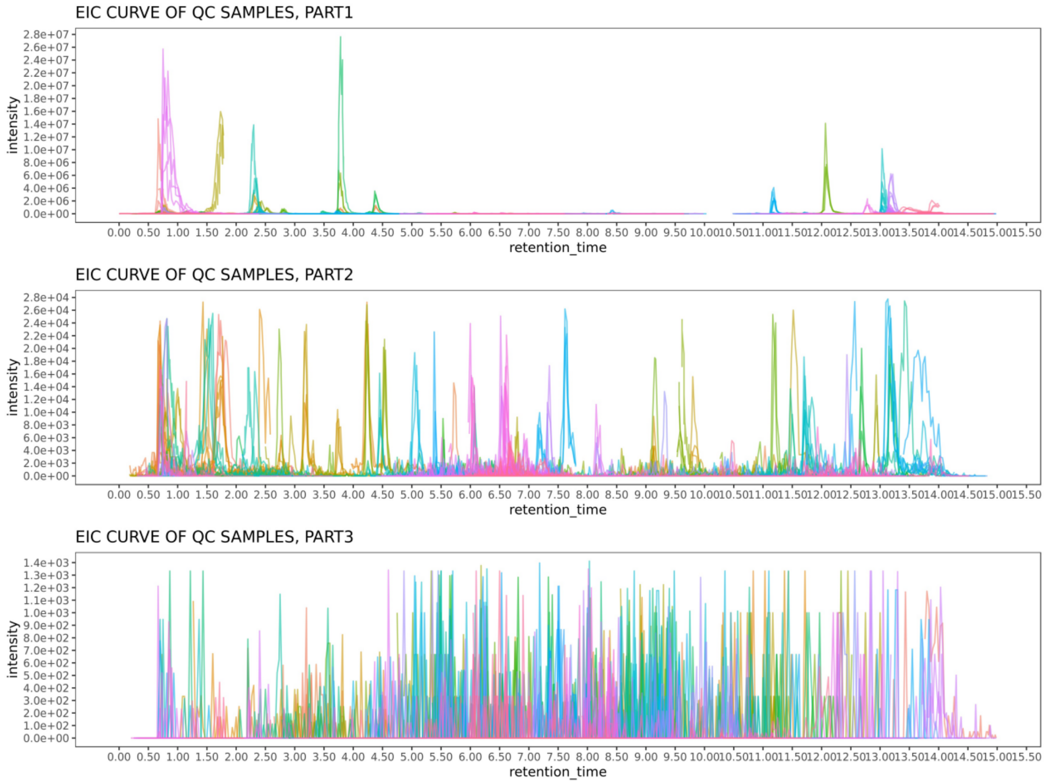

Figure S1 The ion flow in the extraction of quality samples.

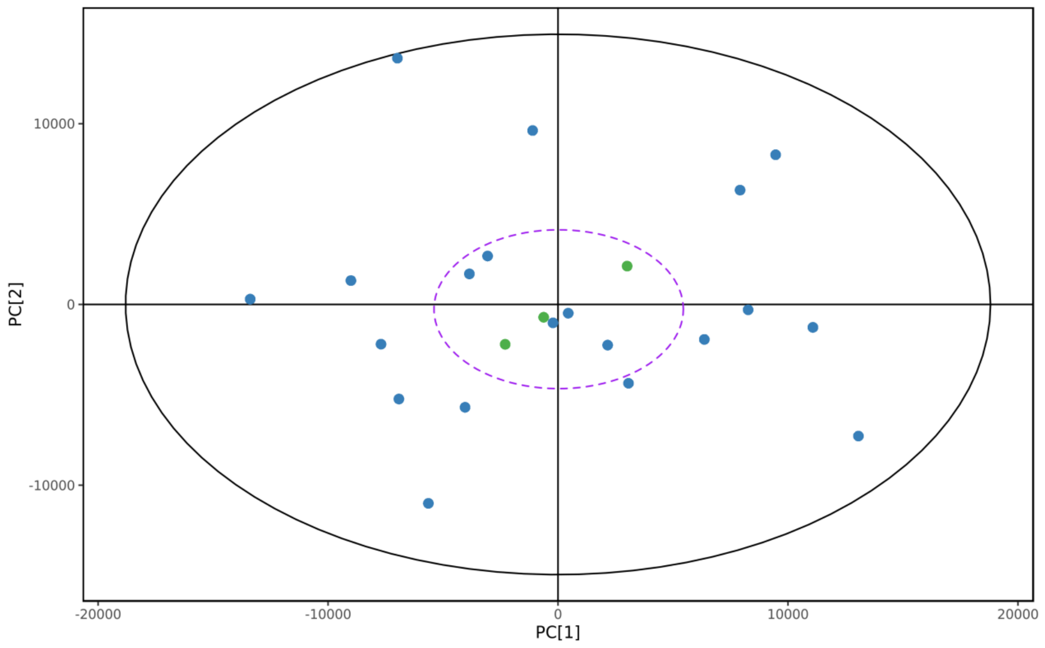

Figure S2 The score of PCA. Green dots represent quality control samples, and blue dots represent experimental samples.

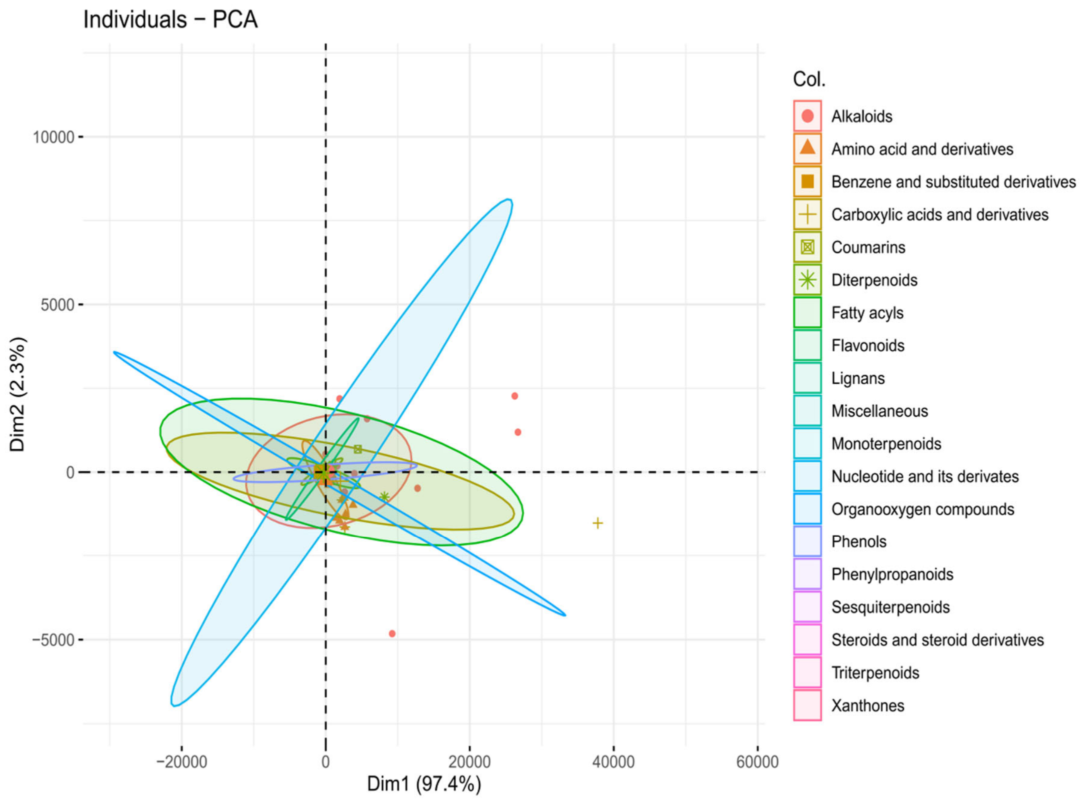

Figure S3 PCA analysis of total compounds from root extracts of invasive *Phytolacca americana*.

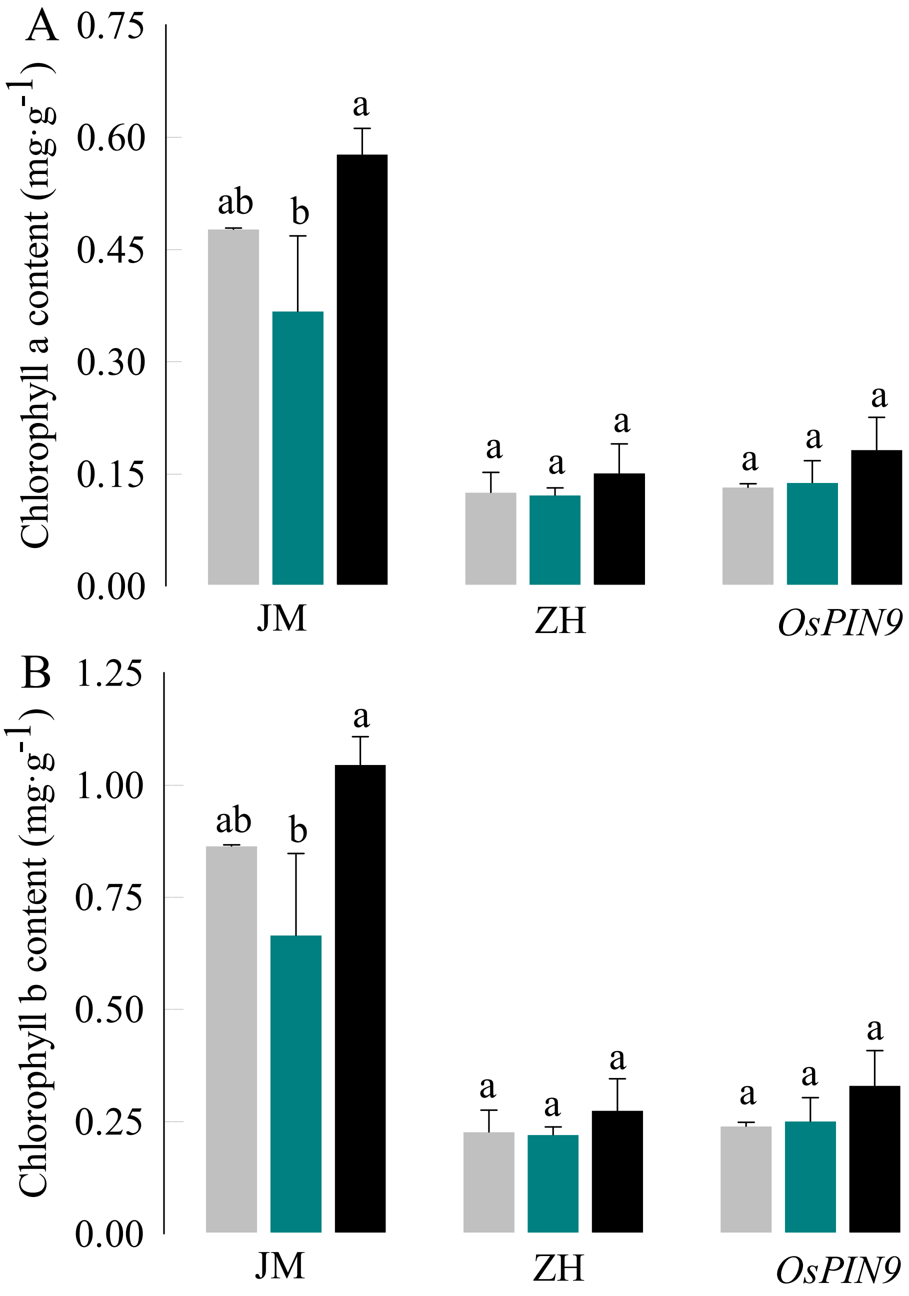

Figure S4 The contents of chlorophyll a (A) and b (B) of three gramineous plants.
